# Carcinomas exhibiting epithelial–mesenchymal transition manifest an M2 macrophage-enriched tumor immune microenvironment

**DOI:** 10.1186/s13058-025-02119-1

**Published:** 2025-10-14

**Authors:** Huang-Chun Lien, Yu-Chia Li, Ruby Yun-Ju Huang, Ko-Chen Chen, Tom Wei-Wu Chen, I-Chun Chen, Li-Ping Hsiao, Ling-Chun Yeh, Yen-Shen Lu

**Affiliations:** 1 Department of Pathology, National Taiwan University Hospital, Taipei, Taiwan; 2 Graduate Institute of Pathology, National Taiwan University, Taipei, Taiwan; 3 School of Medicine, Graduate Institute of Oncology, College of Medicine, National Taiwan University, Taipei, Taiwan; 4 Smart Medicine and Health Informatics Program, International College, National Taiwan University, Taipei, Taiwan; 5 Department of Oncology, National Taiwan University Hospital, Taipei, Taiwan; 6 Department of Medical Oncology, National Taiwan University Cancer Center Hospital, Taipei, Taiwan

## Abstract

**Background:**

Epithelial–mesenchymal transition (EMT) is linked to an immunosuppressive tumor microenvironment (TME). However, direct comparisons of the TME in paired tumor regions with and without EMT in primary tumors—essential for elucidating EMT’s impact on the TME—are still lacking.

**Methods:**

Using Digital Spatial Profiler (DSP) assay and NanoString nCounter Digital Profiling, we analyzed immune-oncology-related markers in the TME of no special type (NST) and paired spindle carcinomatous (SPS) components, the later considered the EMT counterpart of the former, in nine cases of metaplastic breast carcinoma, a type of mammary carcinosarcoma.

**Results:**

We identified macrophage markers as consistently and significantly enriched immune-oncology-related proteins within the TME profiles of SPS components compared to their paired NST counterparts. Additionally, we observed notable enrichment of macrophage-related signatures, M2 macrophage phenotypes, M2 macrophage-inducing cytokines, and M2 macrophage-related genes, as key distinctions in the TME profiles of SPS components relative to NST counterparts. Immunohistochemistry and multiplex immunofluorescence confirmed the presence of M2 macrophages, predominantly characterized by CD14^+^/CD68^+^/CD163^+^ phenotypes, in the SPS components. The in vivo findings were supported by in vitro analysis, which revealed that primary breast cancer cells undergoing spontaneous EMT exhibit enhanced induction of M2 macrophage polarization, with CSF1 contributing to this effect. Despite some heterogeneity, the upregulation of CSF1 and CCL2 likely contributed to the increased presence of M2 macrophages in the SPS components in MpBC cases. This EMT-driven M2 macrophage-enriched TME was also observed in sarcomatous versus carcinomatous components in nonmammary carcinosarcomas across organs, as well as in vimentin-positive versus vementin-negative triple-negative breast cancer, where vimentin expression is associated with EMT.

**Conclusions:**

Given the immunosuppressive role of M2 macrophages, our findings suggest that an EMT-driven, M2 macrophage-enriched TME—a consistent phenomenon across organs—can contribute to immune suppression. This underscores the therapeutic potential of macrophage-targeting strategies and immune checkpoint inhibition in treating aggressive carcinosarcomas characterized by EMT-mediated, M2 macrophage-rich TMEs.

**Supplementary Information:**

The online version contains supplementary material available at 10.1186/s13058-025-02119-1.

## Introduction

The tumor microenvironment (TME) comprises cancer cells and various nonmalignant cells including immune cells within an altered extracellular matrix [1]. TME cells are crucial to cancer progression and metastasis [1, 2, 3]. For instance, tumor-associated macrophages (TAMs) exhibit tumor-promoting effects and contribute to the immunosuppressive nature of the TME [4]. However, the composition and function of the TME vary according to the characteristics of cancer cells [1]. Understanding these complexities is essential for developing effective antitumor therapies that specifically target this environment.

Epithelial–mesenchymal transition (EMT) promotes tumor dissemination by disrupting cell adhesion and inducing cell motility [5, 6]. EMT markers are correlated with immunosuppression in some cancers [7, 8, 9, 10], but genetic heterogeneity among tumors complicates the interpretation of these correlations. Direct comparisons of TMEs in tumor regions with and without EMT are crucial for validating the effects of EMT; however, such comparisons are still limited.

Although EMT plays a major role in carcinoma progression, convincing histopathological evidence of EMT is observed primarily in rare tumor subtypes with a spindle carcinomatous component, such as metaplastic breast carcinoma (MpBC) [11, 12]. MpBC, an aggressive form of breast cancer, typically consists of ductal carcinoma with metaplastic elements, including squamous, spindle, and matrix-producing components [13, 14, 15]. Among these, spindle carcinomatous metaplasia shares histological characteristics with EMT, a correlation consistently supported by transcriptomic and proteomic data [16, 17, 18]. Thus, comparing paired ductal carcinoma and carcinoma with spindle carcinomatous metaplasia within MpBC, which minimizes potential confounding effects stemming from intercase heterogeneity, offers an ideal model to explore EMT’s impact on the TME in a real-world context. In this study, we combined the Digital Spatial Profiler assay—a high-plex spatial profiling technology that enables quantitative measurement of protein expression—with the NanoString nCounter platform for high-plex RNA quantification, along with immunohistochemistry and multiplex immunofluorescence, to comprehensively investigate the influence of EMT on the TME in primary tumors. Our findings provide evidence of EMT-mediated M2 macrophage-enriched TME as a consistent in vivo phenomenon, suggesting the potential utility of macrophage-targeting therapies in treating aggressive carcinomas driven by EMT.

## Materials and methods

### Tumor samples

This study was approved by the Institutional Review Board of National Taiwan University Hospital (approval numbers: 201711051RINC and 202205099RINB). Formalin-fixed, paraffin-embedded (FFPE) surgical specimens were obtained from nine patients with biphasic MpBC featuring invasive carcinoma of no special type (NST) and paired spindle carcinomatous (SPS) components. RNA samples from six cases used for gene expression analysis were collected following the method in our previous study [19]. Additionally, FFPE specimens from 12 patients with carcinosarcoma from various nonmammary organs and from 52 patients with triple-negative breast cancer (TNBC) were included for CD163 immunohistochemical analysis.

## Digital Spatial profiling of immune-oncology-related protein targets and immune cell type markers

In situ profiling of immune oncology protein targets and immune cell markers was performed using the GeoMx DSP assay (NanoString Technologies, Seattle, WA, USA). FFPE sections were stained for immune-oncology-related protein targets and markers of immune cell types, including the immune cell profiling module (β-2 microglobulin, CD3, CD4, CD8, CD11c, CD20, CD45, CD56, CD68, CTLA4, GZMB, HLA-DR, Ki-67, PD-1, and PD-L1), the immune cell typing module (CD14, CD34, CD45RO, CD66b, CD163, and FOXP3), and the immune activation status module (CD25, CD27, CD40, CD44, CD80, CD127, ICOS, and PD-L2), as well as SYTO 13 and two morphological markers, CD45 (2B11 + PD7/26, Novus, CO, USA) and pan-cytokeratin (AE1 + AE3, Novus). The entire slide was imaged, and regions of interest (ROIs) were selected. The GeoMx DSP detected segments with positive CD45 immunofluorescence, which were defined as areas of illumination (AOIs) within each ROI. AOIs were exposed to 385-nm ultraviolet light, and photocleaved oligonucleotides were hybridized to the GeoMx Hyb Code for subsequent processing with the nCounter Prep Station and Digital Analyzer per the manufacturer’s instructions.

## Tumor RNA isolation and gene expression assay

Tumor RNA isolation was performed as previously described [19]. Gene expression profiling (GEP) was conducted using the NanoString PanCancer Immune Oncology 360 panel (PanCancer IO360) on the NanoString nCounter FLEX platform to profile 770 genes related to the tumor-immune interface. Advanced analysis modules were employed for biological pathway enrichments and cell type profiling.

## Bioinformatic analysis

Differentially expressed genes (DEGs) were identified using the R package *limma* or Gene Set Enrichment Analysis (GSEA; Broad Institute, MA, USA). Clustering and visualization of DEG expression levels were calculated using the R packages *pheatmap* and *ggplot2*. Gene sets for GSEA were sourced from the c2.cp, c5.bp, and hallmark collections in the Molecular Signatures Database (MSigDB; version 2022.1). Raw GEP data were deposited in the Gene Expression Omnibus under accession number GSE267035.

## Cell cultures, quantitative reverse transcription polymerase chain reactions, and gene silencing

HE and HM cells, representing paired epithelial- and mesenchymal-predominant primary breast cancer cells, respectively, were established in a previous study [20]. Both cell types were maintained in IH medium containing Dulbecco’s Modified Eagle Medium/F12 (DMEM/F12) (Gibco, Waltham, MA, USA), 2% fetal bovine serum (Gibco), 1% nonessential amino acid (Gibco), 1nM sodium pyruvate, 1 nM nicotinamide (Sigma-Aldrich, Inc., St. Louis, MO, USA), 20 ug/mL insulin (Gibco), 0.5 ug/mL hydrocortisone (Sigma-Aldrich), 10 ng/mL epidermal growth factor (BioLegend, San Diego, CA, USA), 200 μm ascorbic acid (Sigma-Aldrich), adenine (Sigma-Aldrich), primocin (InvivoGen, San Diego, CA, USA), 1 nM estrogen (Sigma-Aldrich), 100 nM retinoic acid (Cayman Chemical, Ann Arbor, MI, USA), and 0.2 pg/mL triiodothyronine (Sigma-Aldrich). MDA-MB-231 and Hs 578T breast cancer cells were maintained in DMEM/F12 (Gibco) and DMEM (Gibco), respectively, each supplemented with 10% fetal bovine serum (Gibco). MCF10A breast cells were maintained in DMEM/F12 supplemented with 5% horse serum (Gibco), 100 ng/ml cholera toxin (Sigma-Aldrich), 20 ng/ml epidermal growth factor (BioLegend), 500 ng/ml hydrocortisol (Sigma-Aldrich) and 10 ug/ml insulin (Gibco). For induction of EMT, the MCF-10 A cells were treated with recombinant human TGF-β1 (R&D Systems, Minneapolis, MN, USA) at a concentration of 5 ng/ml for 120 h before analysis. Human monocytic THP-1 cells were cultured in Roswell Park Memorial Institute (RPMI) medium. HE or HM cells were incubated in serum-free RPMI medium before being collected and centrifuged, after which the conditioned medium was applied to M0 macrophages derived from THP-1 cells treated with phorbol-12-myristate-13-acetate (PMA) for 24 h [21]. After 72 h, RNA was extracted from the macrophages using REzolTM C&T (PROtech, Taipei, Taiwan) and reverse transcribed into cDNA (Superscript IV reverse transcriptase; Thermo Fisher Scientific, Waltham, MA, USA). Quantitative reverse transcription polymerase chain reaction (qRT-PCR) for M1 and M2 macrophage markers was conducted on an ABI PRISM 7900 Sequence Detection System (Applied Biosystems, Foster City, CA, USA) using the SYBR Green method. Quantitative values were calculated using the 2^−∆∆Ct^ method, with target gene measurements in all samples normalized to GAPDH. Lentiviral shRNA particles targeting CSF1 (TRCN0000262776 and TRCN0000282422) and ZEB1 (TRCN000017565) were obtained from the RNAi Core Laboratory of the Academia Sinica (Taipei, Taiwan). With an shRNA vector targeting lacZ (pLKO.1-shLacZ) used as the control. HM cells were infected with lentiviruses for 24 h and selected in medium containing 1.5 mg/mL puromycin (Gibco) for one week prior to further analysis. Primer sequences are listed in Supplementary Table S1.

### Western blotting and enzyme-linked immunosorbent assay (ELISA)

For Western blotting, proteins from cell lysates were separated on a sodium dodecyl sulfate polyacrylamide gel and transferred onto nitrocellulose membranes (Millipore, Bedford, MA, USA). The antibodies used included anti-E-cadherin (RM-2100-S, Thermo Fisher), anti-Vimentin (vim; GTX100619, GeneTex, Irvine, CA, USA), anti-N-cadherin (sc-7939, Santa Cruz Biotech., Dallas, Taxis, USA), anti-CSF1 (2D10, Novus), and anti-CD163 (MA5-11458). CSF1 protein levels in the medium were measured using an ELISA kit (EK0444, Boster Biological Technology, Pleasanton, CA, USA) according to the manufacturer’s instructions.

## Immunohistochemistry

Immunohistochemical staining was performed using a Ventana iVIEW DAB detection kit with the autoimmunostainer (Ventana BenchMark). Staining for CD163 (MA5-11458, Thermo Fisher), CD68 (Ab192847, Abcam, Cambridge, United Kingdom), CD14 (HPA119197, Sigma-Aldrich), and vim (Clone V9, Ventana Medical Systems) was conducted according to the manufacturer’s protocols. Labeling was detected using the Ultraview DAB Detection Kit (Ventana Medical Systems) as instructed by the manufacturer. Staining results were analyzed using StrataQuest v6.0 (TissueGnostics, Vienna, Austria) or manually by counting the average number of positive cells per 0.96 μm².

## Multiplex immunofluorescence staining

FFPE tissue sections were analyzed using the Polaris system (PerkinElmer, Waltham, MA, USA) with a customized Opal 4-color panel targeting CD14 (HPA119197, Sigma-Aldrich), CD68 (Ab192847, Abcam), CD163 (MA5-11458, Thermo Fisher), and CD45 (Ab10558, Abcam) following the manufacturer’s instructions.

### Statistical analysis

Data processing, analysis, and plotting were performed using R software and GraphPad Prism version 6 for Windows (GraphPad Software). *P* values were calculated using paired *t*-tests for paired samples and standard *t*-tests for unpaired samples.

## Results

### DSP assay reveals macrophage enrichment in EMT-associated TME of MpBCs

**Fig. 1:**
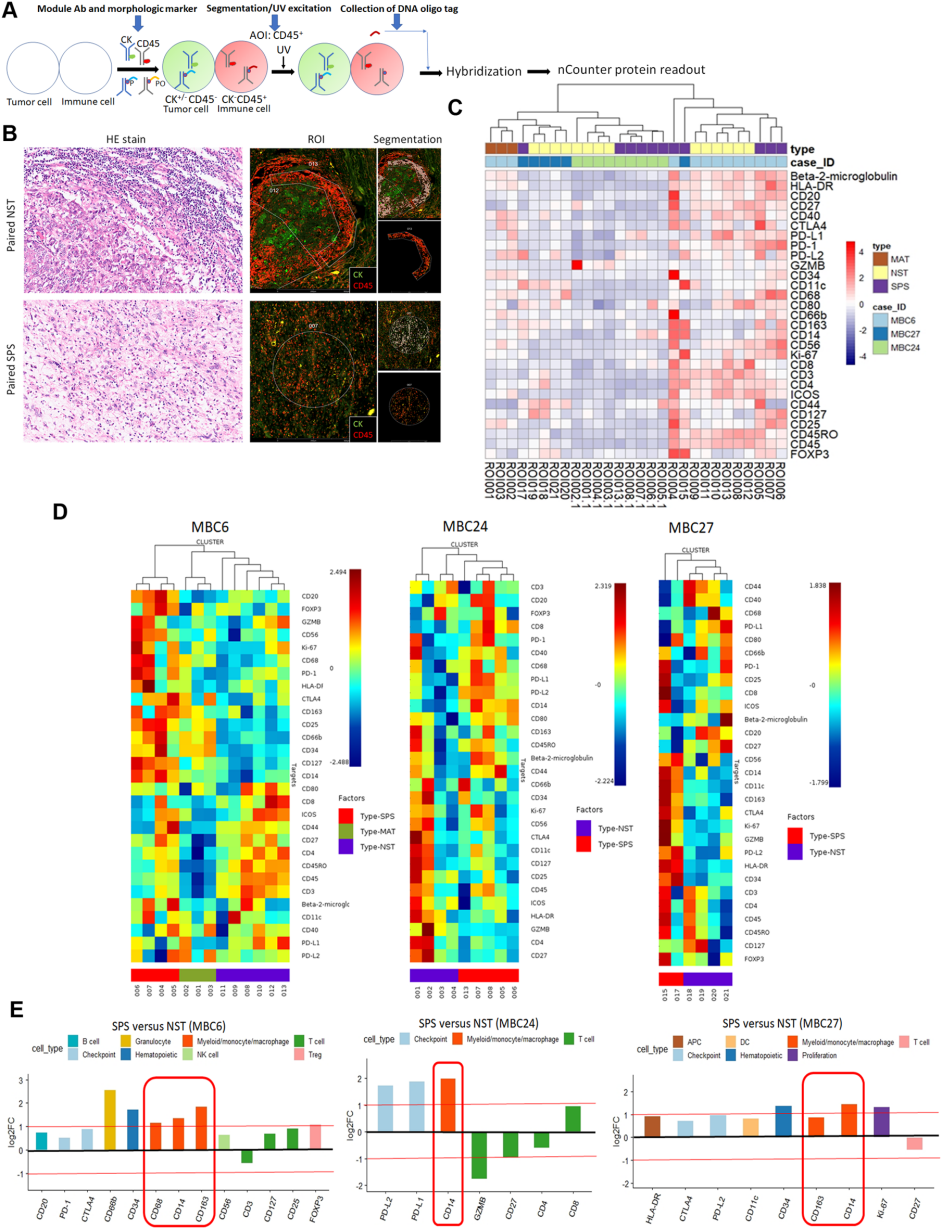
The Digital Spatial Profiler (DSP) assay reveals an enrichment of macrophages in the tumor microenvironment of spindle carcinomatous (SPS) components compared to paired no special type (NST) components across all three metaplastic breast carcinoma (MpBC) case. **(A)** Schematic of the DSP assay. P, photocleavable linker; PO, photocleavable oligo; AOI, area of illumination; CK, pan-cytokeratin. **(B)** Representative histomorphology showing region of interest (ROI) and AOI segmentation using morphologic markers CK and CD45 in paired NST and SPS components of case MBC6. **(C)** Heatmap displaying unsupervised clustering of ROIs from the three MpBC cases analyzed by DSP. **(D)** Heatmaps showing unsupervised clustering of ROIs for the three MpBC cases analyzed by DSP. **(E)** Bar plots presenting differentially expressed immune-oncology-related protein markers in SPS versus paired NST components across the three MpBC cases, with macrophage markers (indicated by circles) consistently upregulated in all cases

We utilized the GeoMx DSP assay to analyze immune-oncology-related protein markers with spatial resolution within the EMT-associated TME, focusing on immune cell profiling, typing, and activation in both NST and paired SPS components, the latter being considered the EMT counterpart of the former, across three MpBC cases (Fig. 1A-B, Supplementary Fig. 1). The heatmap indicated inter-case heterogeneity but revealed distinct intra-case clustering of protein expression among the NST and SPS components (Fig. 1C). Further analysis demonstrated clear clustering of the SPS components, distinguishing them from the paired NST components in all cases (Fig. 1C-D). The matrix-producing component in one MpBC case did not cluster with its paired components. Among the markers highly enriched in the SPS compared to the paired NST components—defined as log_2_FC ≥ 1 or ≤ -1—monocyte/macrophage markers (CD14, CD68, and CD163) were the only ones consistently observed across all cases. Specifically, CD68, CD14, and CD163 were detected in MBC6, while CD14 was observed in MBC24 and MBC27 (Fig. 1E, Supplementary Table S2). In contrast, the granulocyte marker CD66b and the regulatory T cell (Treg) marker FOXP3 were highly expressed in the SPS only in MBC6, and the immune checkpoint proteins PD-L1 and PD-L2 were highly expressed exclusively in MBC24. These findings underscore the consistent enrichment of monocyte/macrophage markers in the EMT-associated TME.

### Digital profiling reveals enrichment of macrophage-related signatures, M2 macrophage phenotypes, M2 macrophage-inducing cytokines, and M2 macrophage-related genes in the EMT-associated TME of MpBCs

**Fig. 2:**
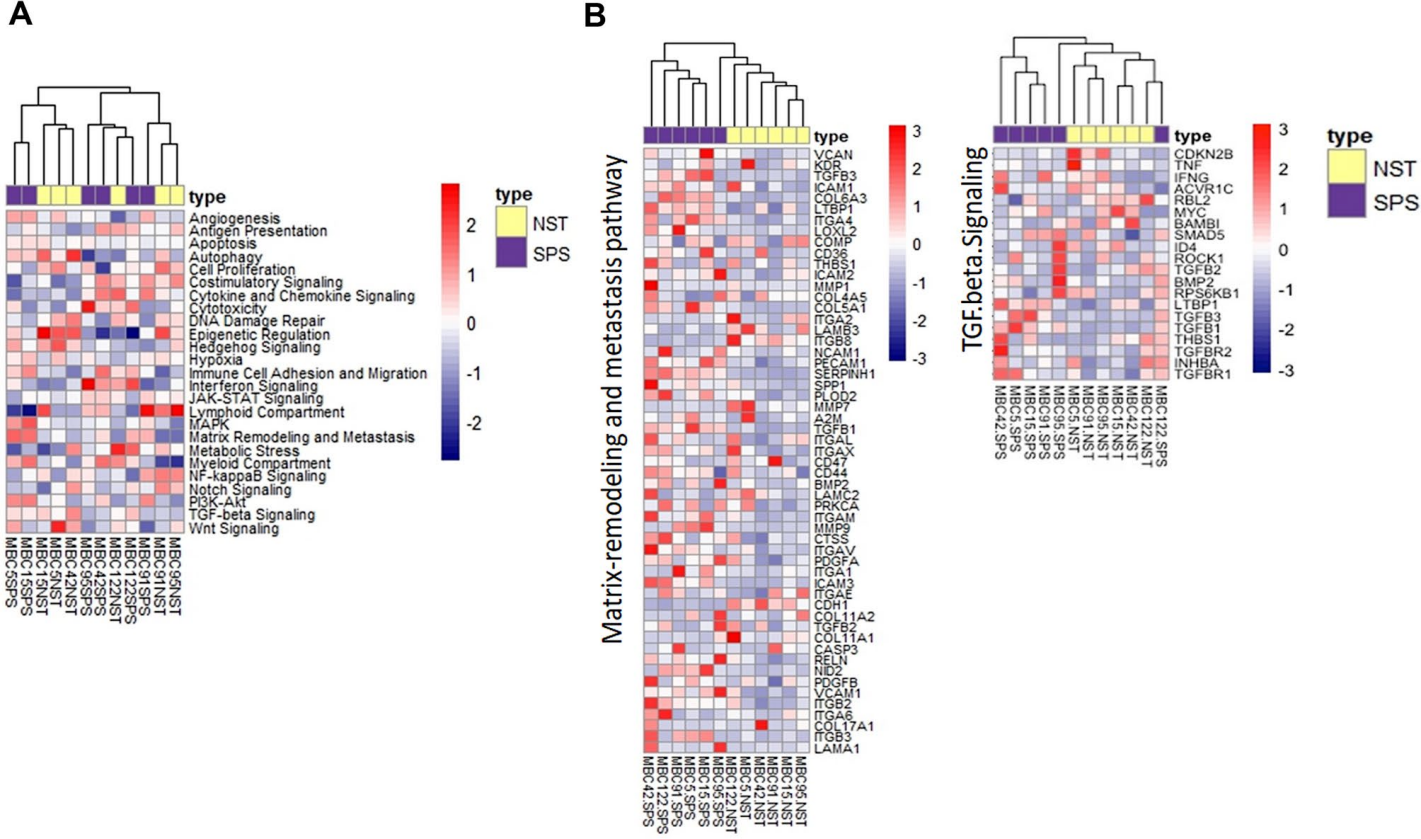
Heatmaps depicting NanoString-defined biological pathways based on the IO360 panel’s 770 genes related to the tumor-immune interface for both spindle carcinomatous (SPS) and paired no special type (NST) components across six additional metaplastic breast carcinoma (MpBC) cases (MBC5, 15, 42, 91, 95, and 122). **(A)** Heatmap illustrating 25 NanoString IO360-defined biological pathways in SPS and paired NST components of the six MpBC cases. **(B)** Heatmaps depicting biological pathways associated with matrix remodeling, metastasis, and TGF-β signaling were created for the SPS and paired NST components across the six MpBC cases

**Fig. 3:**
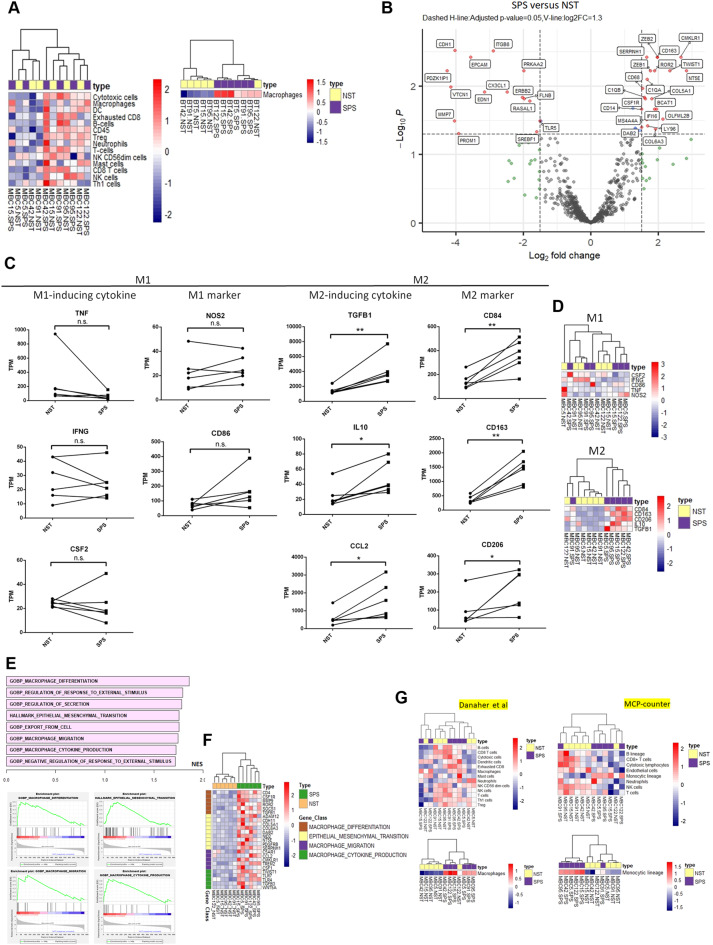
Differentially expressed IO360-defined immune cell type scores, genes, and related functions, in the tumor microenvironment (TME) of spindle carcinomatous (SPS) versus paired no special type (NST) components across six cases of metaplastic breast carcinoma (MpBC). **(A)** Heatmaps illustrating NanoString IO360-defined immune cell type scores (left panel) and macrophage-specific scores (right panel) observed in the SPS and paired NST components across the six MpBC cases. **(B)** Volcano plot depicting the differentially expressed genes in SPS versus NST components across the six cases of MpBC. The x-axis represents the log_2_ fold change (SPS/NST), while the y-axis depicts the − log_10_ adjusted *p* value. H-line, horizontal line; V-line, vertical line; log_2_ FC, log_2_ fold change. **C**,** D.** Dot-line graph (**C**) and heatmaps (**D**) depicting the abundant expression of genes for M1 and M2 macrophage-inducing cytokines, as well as M1 and M2 macrophage cell markers, observed in the SPS and paired NST components across the six MpBC cases. n.s. not significant, *p < 0.05, **p < 0.01, paired-t test. **E.** Bar plot (upper panel) showing the top enriched biological processes and hallmark molecular pathways in SPS versus NST components, as determined by gene set enrichment analysis (GSEA). NES, normalized enrichment score. Representative enrichment plots are shown in the lower panel. **F.** Heatmaps depicting leading genes associated with biological processes and hallmark molecular pathways. **G.** Heatmaps illustrating various immune cell type scores (upper panel) and macrophage-specific scores (lower panel) as defined by two external datasets: Danaher et al. (PMID 28239471) and Becht et al. (MCP-counter, PMID 26994146) in the SPS and paired NST components across the six MpBC cases. MCP-counter, microenvironment cell populations counter

To further investigate the in vivo impact of EMT on the TME, we analyzed gene expression in paired SPS and NST components from six additional MpBC cases. This analysis involved digital profiling of the IO360 panel-defined 770 genes and related biological pathways relevant to the tumor-immune interface. Among the 25 biological pathways assessed, only the matrix-remodeling and metastasis pathway and the tumor growth factor beta (TGF-β) signaling pathway effectively differentiated the SPS components from the NST components (Fig. 2A-B, Supplementary Fig. S2). The upregulation observed in SPS components aligns with EMT features [22, 23]. Besides, among the 14 IO360-defined immune cell types, only macrophage cell type scores distinguished the SPS components from the NST components (Fig. 3A, Supplementary Table S3). Analysis of differentially expressed genes revealed upregulation of *ZEB1*, *ZEB2* and *TWIST1*, and downregulation of *CDH1* and *EPCAM*, in SPS compared to NST components, consistent with EMT features in SPS metaplasia [16, 17, 18, 19] (Fig. 3B). Additionally, the SPS components exhibited *CD14*, *CD68*, *CD163*, and *CSF1R* upregulation—markers indicative of an M2 macrophage phenotype [24, 25, 26, 27, 28]—as well as increased expression of M2 macrophage-associated genes (*C1QA*, *C1QB*, *DAB2*, *MS4A4A* and *OLFML28*; Fig. 3B). These findings align with the observed enrichment of M2 macrophage phenotypes and M2 macrophage-inducing cytokines in the SPS compared to NST components (Fig. 3C-D, Supplementary Table S4), along with pathways involving macrophage differentiation, migration, and cytokine production ranking among the top SPS-enriched pathways (Fig. 3E, F). The enrichment of macrophage cell types was further validated using two external immune cell gene sets [29, 30] (Fig. 3G, and Supplementary Table S5 and S6). Overall, these results demonstrate M2 macrophage enrichment in the EMT-associated TME.

### Validation of M2 macrophage enrichment in the EMT-associated TME using immunohistochemistry and multiplex immunofluorescence staining

**Fig. 4:**
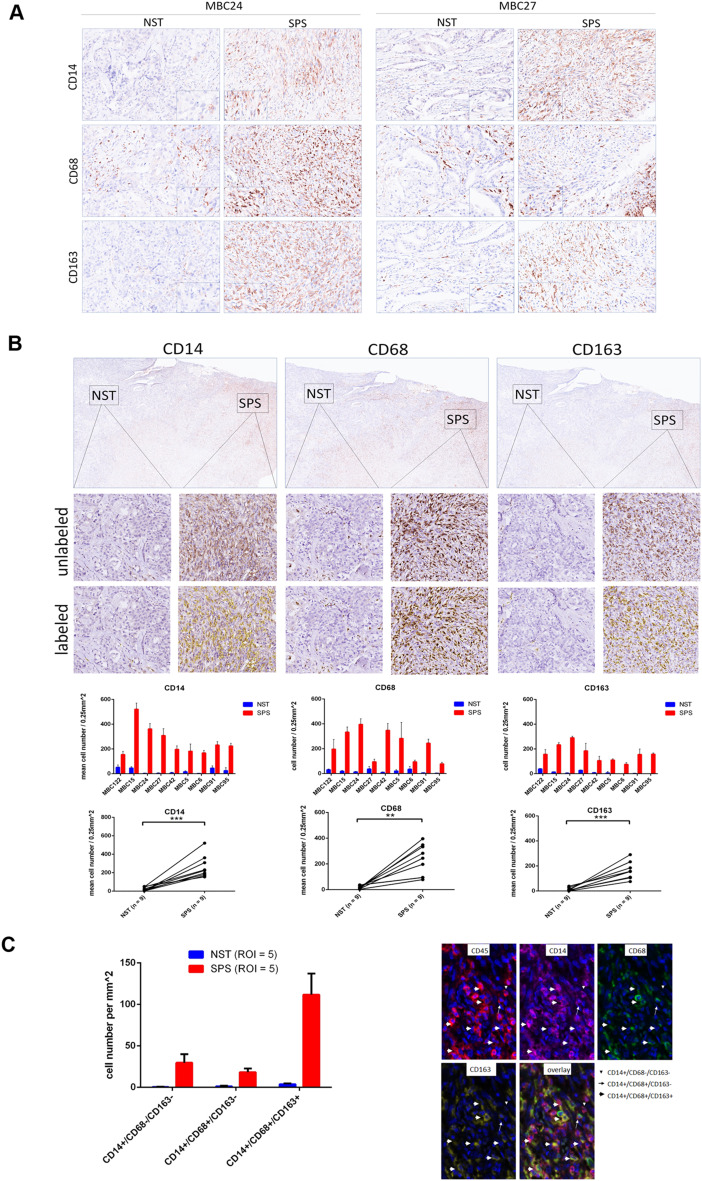
Immunohistochemical and multiplex immunofluorescence staining performed to examine macrophage markers CD14, CD68, and CD163 in both the spindle carcinomatous (SPS) and paired no special type (NST) components of metaplastic breast carcinoma (MpBC) cases. **(A)** Immunochemistry for CD14, CD68, and CD163 staining in the SPS and paired NST components in two representative MpBC cases. **(B)** Quantitative analysis of immune cells stained for CD14, CD68, and CD163 conducted using StrataQuest in the SPS and paired NST components for a representative MpBC case (MBC24). Representative immunohistochemistry images for CD14, CD68, and CD163 (upper panel), as well as images of stained cells with and without StrataQuest labeling (middle panels) in SPS and paired NST components. Bar plots and dot-line graphs (lower panels) present the quantitative results, expressed as means ± standard deviations of cell counts per 0.25 mm², comparing SPS to paired NST components across nine MpBC cases. **p < 0.01, ***p < 0.001, paired *t* test. **(C)** Bar plots (left panel) show the means ± standard deviations of cells co-stained for CD14, CD68, and CD163 per 0.25 mm², as indicated by the opal multicolor panel, in the SPS and paired NST components for a representative MpBC case. Representative images of the multiplex immunofluorescence staining are shown in the right panel

To validate M2 macrophage enrichment in the EMT-associated TME, we performed immunohistochemical staining for CD14, CD68, and CD163 on paired SPS and NST components from nine cases of MpBC. Quantitative analysis revealed significantly increased staining for all three markers in the SPS components compared to the NST components across all cases (Fig. 4A-B). Furthermore, multiplex immunofluorescence staining in a representative MpBC case demonstrated that CD14+/CD68+/CD163 + cells—indicative of M2 macrophages—were the predominant cell type in the SPS components, significantly outnumbering those in the paired NST components (Fig. 4C). These results confirm the enrichment of M2 macrophages in the SPS components relative to the paired NST components in MpBC.

### Primary breast cancer cells undergoing EMT exhibit enhanced induction of M2 macrophage polarization

**Fig. 5:**
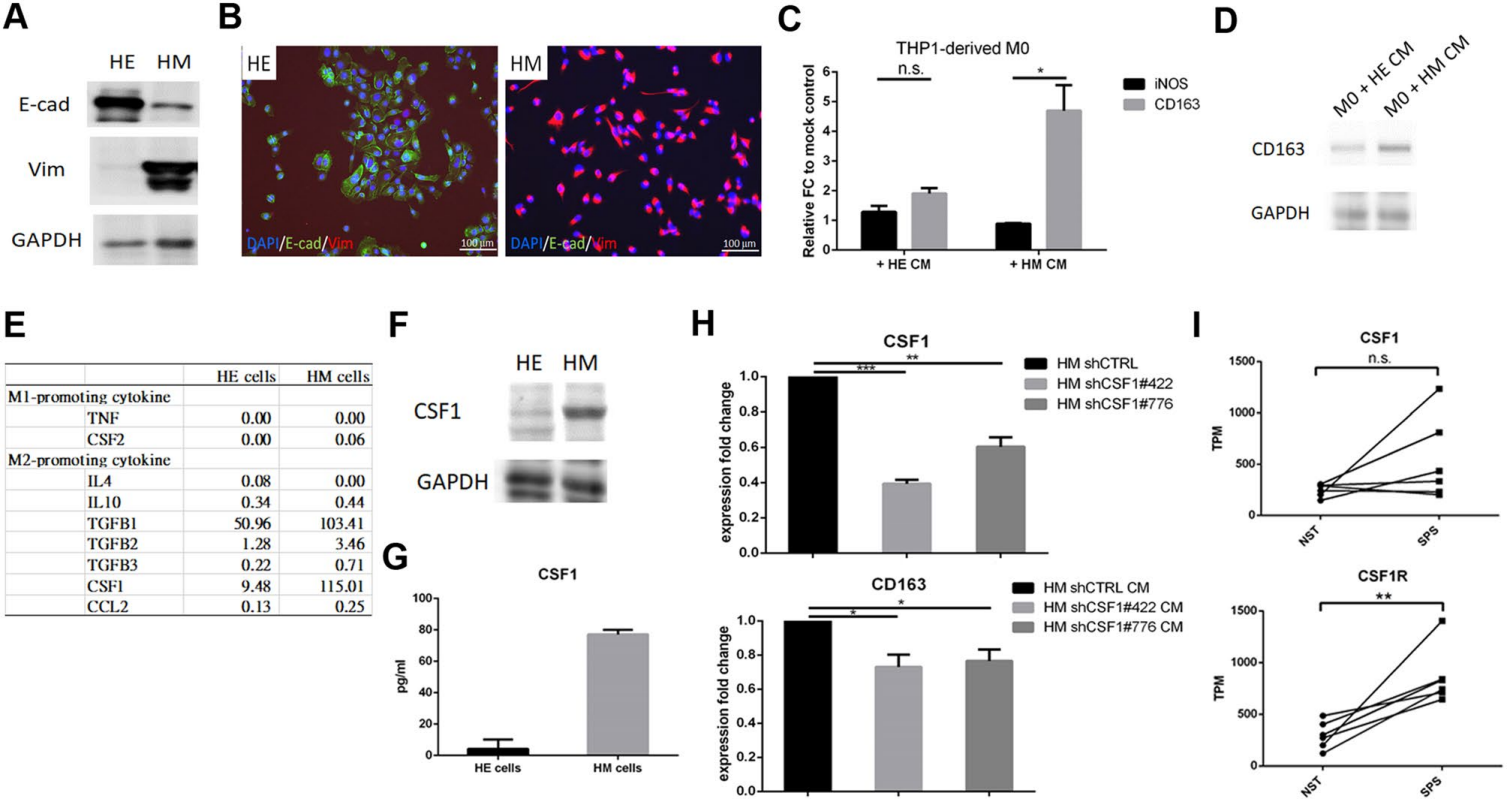
Primary breast cancer cells undergoing epithelial–mesenchymal transition (EMT) more effectively promote M2 macrophage polarization. **A**,** B.** Western blot (**A**) and immunofluorescence staining (**B**) showing the dominant expression of the epithelial marker E-cadherin (E-cad) in epithelial-predominant HE cells and the mesenchymal marker vimentin (Vim) in mesenchymal-predominant HM cells. The HM cells represent the EMT counterparts of the HE cells. **C.** Bar plots displaying the fold changes in M1 macrophage marker *iNOS* and M2 macrophage marker *CD163*, measured by quantitative real-time polymerase chain reaction (qRT-PCR), in THP1-derived M0 macrophages treated with conditioned medium (CM) from HE or HM cells for 72 h, relative to a mock control. FC, fold change; CM, conditioned medium; n.s., not significant. *p < 0.05, *t* test. **D.** Western blot showing expression of the M2 macrophage marker CD163. **E.** RNA sequencing results indicating gene expression levels, of M1- and M2-promoting cytokines in HE and HM cells, presented as transcripts per million (TPM). **F**,** G.** Western blot (**F**) and enzyme-linked immunosorbent assay (ELISA) (**G**) results showing CSF1 expression in cell lysates and culture medium, respectively, from HE and HM cells. **H.** Bar plots presenting qRT-PCR results: fold changes in *CSF1* RNA levels in HM cells with *CSF1* knockdown versus control (upper panel) and fold changes in *CD163* RNA levels in THP-1-derived M0 macrophages treated with conditioned medium from *CSF1*-knockdown HM cells versus control (lower panel). *p < 0.05, **p < 0.01, ***p < 0.001, *t* test. **I.** Dot-line plots illustrating gene expression levels of *CSF1* and *CSF1R* in the spindle carcinomatous (SPS) and paired no special type (NST) components across six MpBC cases. n.s., not significant, **p < 0.01, paired-t test

We previously established paired epithelial-predominant HE cells and mesenchymal-predominant HM cells from a primary breast cancer culture, with HM cells representing the spontaneous EMT counterpart of HE cells [20] (Fig. 5A, B, Supplementary Fig. S3). To validate the EMT-driven M2 macrophage polarization observed in vivo and investigate underlying mediators, we assessed whether the HM cells could more effectively induce M2 macrophage polarization than HE cells. THP-1-derived M0 macrophages showed substantially higher RNA levels of the M2 macrophage marker *CD163*—but not the M1 marker *iNOS*—when treated with HM-conditioned medium compared to HE-conditioned medium (Fig. 5C). Western blot analysis results confirmed this M2 macrophage polarization (Fig. 5D, Supplementary Fig. S4). Using RNA sequencing, we explored M2 macrophage-promoting cytokines in HM and HE cells and found *CSF1* expression to be substantially higher in HM cells than in HE cells (Fig. 5E). CSF1 levels were also elevated in both cell lysates and conditioned medium from HM cells (Fig. 5F, G, Supplementary Fig. S4). Additionally, CSF1 knockdown in HM cells reduced CD163 expression in THP-1-derived M0 macrophages, indicating a reversion of HM-induced M2 macrophage polarization and supporting CSF1’s role in this process (Fig. 5H) [31]. To further confirm the role of EMT in promoting M2 macrophage polarization, we knocked down ZEB1 in mesenchymal-predominant HM cells and in two mesenchymal-type breast cancer cell lines, MDA-MB-231 and Hs 578T. As shown in Supplementary Fig. S5A–I, ZEB1 knockdown in all three cell types suppressed mesenchymal characteristics, as indicated by reduced vimentin expression, along with a concomitant decrease in CSF1 expression. Consistently, conditioned medium from ZEB1-knockdown cells reduced M2 polarization of THP1-derived M0 macrophages compared to CM from control cells. In addition, TGF-β treatment of MCF10A breast epithelial cells induced EMT and upregulated CSF1, which in turn enhanced M2 polarization of THP1-derived M0 macrophages (Fig. S5J-L). These in vitro findings align with the EMT-driven M2 macrophage enrichment observed in vivo and underscore CSF1’s role in promoting EMT-driven M2 macrophage polarization. Notably, the upregulation of *CSF1* and *CSF1R* in the SPS components relative to paired NST components in several MpBC cases supports this observation (Fig. 5I).

### Selective enrichment of M2 macrophages in sarcomatous versus carcinomatous components of nonmammary carcinosarcomas and in vim-positive versus vim-negative TNBC

**Fig. 6:**
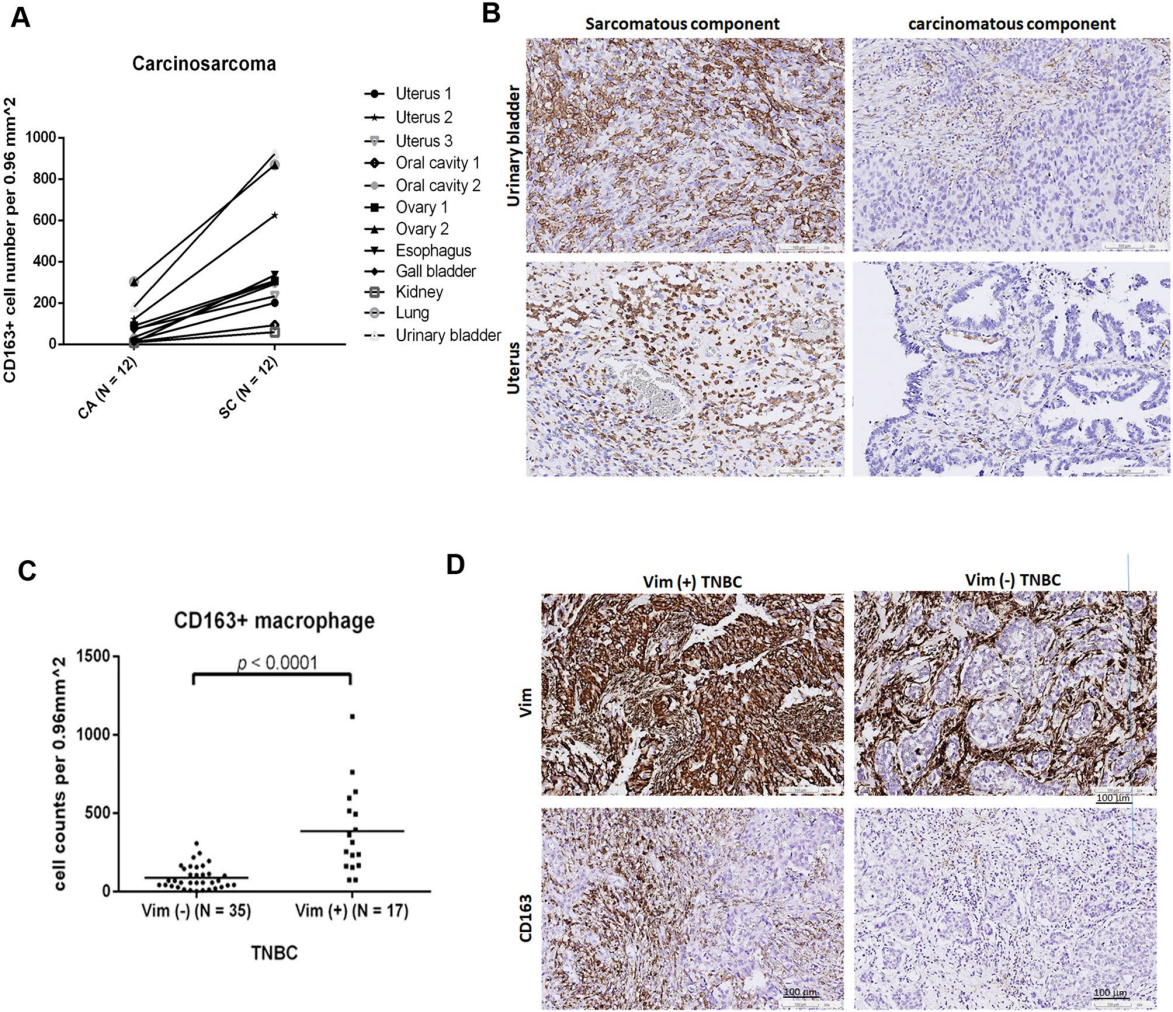
Increased presence of CD163-positive M2 macrophages observed in the sarcomatous compared to paired carcinomatous components in nonmammary carcinosarcomas, and in Vimentin (Vim)-positive compared to Vim-negative triple-negative breast cancer (TNBC) cases. **(A)** Dot-line plot showing the number of CD163-positive M2 macrophages per 0.96 mm^2^ in both the carcinomatous (CA) and paired sarcomatous (SC) components across 12 cases of nonmammary carcinosarcoma from various organs. **(B)** Immunohistochemistry showing CD163-positive macrophage staining in the sarcomatous and carcinomatous components of representative cases of carcinosarcoma involving the urinary bladder and uterus. **(C)** Dot plot illustrating the number of CD163-positive M2 macrophages per 0.96 mm^2^ in TNBCs with and without Vim expression. **(D)** Immunohistochemistry demonstrating CD163-positive macrophage staining in representative cases of Vim-positive and Vim-negative TNBC

We further examined whether the EMT-driven enrichment of M2 macrophages observed in MpBC, a mammary carcinosarcoma, also occurred in carcinosarcomas from other organs. Consistent with the finding in MpBC, M2 macrophages were universally more abundant in the sarcomatous components compared to the carcinomatous components in carcinosarcomas across various organs (Fig. 6A-B). Additionally, given the association between vim expression and EMT in TNBC [32], we assessed this relationship in TNBC cases and found significantly higher numbers of M2 macrophages in vim-positive versus vim-negative TNBC (Fig. 6C-D). These findings provide in vivo evidence that EMT-driven M2 macrophage enrichment is a widespread phenomenon across multiple organ systems.

## Discussion

Carcinosarcoma, found in both mammary and nonmammary tissues is characterized by the coexistence of carcinomatous and various sarcomatous metaplastic components, with the spindle metaplastic element considered the EMT counterpart of the carcinomatous component. Despite its rarity, carcinosarcoma offers a valuable model for studying EMT in real-world settings [11]. In this study, we analyzed immune-oncology-related proteins, immune cell markers, and 770 genes related to the tumor-immune interface in NST and paired SPS components across nine MpBC cases. Notably, we identified an enrichment of M2 macrophage phenotypes, M2 macrophage-inducing cytokines, and M2 macrophage-related genes—such as *C1QA* and *C1QB* [24, 25], *DAB2* [26], *MS4A4A* [27], and *Olfr78* [28]—as key distinctions in the TME profiles of SPS compared to NST counterparts. Pathways associated with macrophage differentiation, migration, and cytokine production were also enriched in the SPS components. Immunohistochemistry and multiplex immunofluorescence confirmed M2 macrophage enrichment, with CD14^+^/CD68^+^/CD163^+^ M2 macrophages predominantly present in the SPS components versus NST components. This M2 macrophage-enriched TME was also observed in sarcomatous versus carcinomatous components of carcinosarcomas from various nonmammary organs. Additionally, we found increased M2 macrophage presence in vim-positive TNBCs relative to vim-negative cases, with vim expression associated with EMT [32]. These findings suggest that EMT-mediated, M2-macrophage-enriched TMEs may represent a consistent in vivo phenomenon, a notion further supported by similar associations in breast cancer mouse models [33] and head-and-neck squamous cell carcinoma cell lines [34].

To validate our in vivo findings and explore mediators, we conducted an in vitro study using a previously established pair of genetically unperturbed primary breast cancer cells: epithelial-predominant HE cells and mesenchymal-predominant HM cells, with the latter representing the EMT counterpart of HE cells [20]. HM cells induced significantly enhanced M2 macrophage polarization compared to HE cells, confirming EMT-driven M2 macrophage enrichment in the TME. The elevated expression of CSF1 in HM cells, along with the reversal of M2 macrophage polarization following CSF1 knockdown, supports CSF1’s known role in promoting M2 macrophage polarization [31], and highlights its importance in EMT-driven M2 macrophage polarization. Consistently, *CSF1* expression was also higher in SPS components than in NST components in certain MpBCs. Additionally, *CCL2*, a crucial factor for M2 macrophage recruitment, was notably elevated in SPS components but showed minimally expression in both HE and HM cells (Figs. 3C and 5E). These findings confirm EMT-driven M2 macrophage enhancement in the TME and suggest CSF1 and CCL2 as key mediators, although there may be heterogeneity among the mediators involved.

MpBC is notably more aggressive and resistant to chemotherapy than invasive ductal carcinoma [12, 35, 36]. Studies indicate that prognosis of MpBCs varies by metaplastic subtype, with spindle cell carcinoma (spindle metaplasia) being particularly aggressive [37, 38]. M2 macrophages are known to promote tumor growth, metastasis, and resistance to chemotherapy and immunotherapy [39], suggesting that EMT-driven M2 macrophages may contribute to the poor treatment outcomes and prognosis seen in both mammary and nonmammary carcinomas with spindle carcinomatous metaplasia. Given the crucial role of CSF1R in TAM accumulation and migration [40], and the established strategy of blocking the CSF-1/CSF-1R axis to inhibit the recruitment and polarization of M2-like TAMs in TAM-targeting immunotherapy [39, 41, 42], along with the observed higher PD-L1 expression tumor cell in spindle compared to epithelial tumor cells [43], our findings of EMT-induced CSF1 upregulation and elevated CSF1/CSF1R expression in SPS components may have important therapeutic significance. Specifically, TAM-targeting therapies, immune checkpoint inhibitors, and EMT-targeting agents such as anti-TGF-β agents [44] may be promising treatment approaches for aggressive carcinosarcomas with an EMT-driven, M2-macrophage-rich TME.

CD163, an M2 macrophage marker, is significantly enriched in TNBC tissues [45]. Elevated CD163 RNA and protein levels are correlated with worse survival outcomes in TNBC cases [45, 46], highlighting the detrimental impact of M2 macrophages in this breast cancer subtype. Vim expression in breast cancer is associated with EMT in both in vitro and in vivo studies [47]. In MpBC, vim is expressed in nearly all SPS components, compared to only 12–40% in the NST areas [20, 43]. In TNBC, vim expression correlates with CD146—an EMT inducer—and is associated with the CD44⁺/CD24⁻ stem cell phenotype, which is closely tied to EMT. Our finding of significantly higher numbers of CD163⁺ M2 macrophages in vim-positive versus vim-negative TNBC tissues is consistent with the enrichment of M2 macrophages in SPS compared to paired NST components in both mammary and non-mammary carcinosarcomas, highlighting the role of EMT in promoting an M2 macrophage–rich microenvironment. Our finding of a significantly higher number of CD163^+^ M2 macrophages in vim-positive compared to vim-negative TNBC tissues aligns with the enrichment of M2 macrophages in the SPS versus paired NST components in mammary and nonmammary carcinosarcomas, underscoring the role of EMT in promoting M2 macrophages. Given the poor prognosis associated with M2 macrophages in TNBC [45, 46], our findings suggest that incorporating TAM-targeting therapy may be beneficial for treating TNBC, particularly in vim-positive cases with elevated M2 macrophage levels.

In summary, this study identified M2-macrophage-related genes and proteins as the primary differences in the TME profiles between SPS and NST components in MpBC. This EMT-driven enrichment of M2 macrophages extends to nonmammary carcinosarcomas and TNBC. Our in vivo findings, supported by in vitro results, demonstrate that breast cancer cells undergoing spontaneous EMT effectively induce M2 macrophage polarization, with CSF1 playing a key role in this process. Although some variability exists, the upregulation of CCL2 and CSF1 likely contributes to the enhanced M2 macrophage presence in SPS components. Overall, our findings provide strong evidence that EMT-driven M2 macrophage enrichment in the TME is a consistent phenomenon across organs, suggesting that macrophage-targeting therapies could be viable strategies for treating aggressive carcinosarcomas with EMT-driven, M2-macrophage-rich TMEs.

## Supplementary Information

Below is the link to the electronic supplementary material.

Supplementary Material 1

Supplementary Material 2

Supplementary Material 3

Supplementary Material 4

Supplementary Material 5

Supplementary Material 6

Supplementary Material 7

Supplementary Material 8

Supplementary Material 9

Supplementary Material 10

Supplementary Material 11

## Data Availability

No datasets were generated or analysed during the current study.

## Abbreviations

AOI: Area of illumination
C1QA: Complement C1q A chain
C1QB: Complement C1q B chain
CCL2: C-C Motif Chemokine Ligand 2
CDH1: Cadherin 1
CSF1: Colony stimulating factor 1
CSF1R: Colony stimulating factor 1 receptor
DAB2: DAB adaptor protein 2
DEG2: Differentially expressed genes
DSP: Digital spatial profiler
ELISA: Enzyme-linked immunosorbent assay
EMT: Epithelial-mesenchymal transition
EPCAM: Epithelial cell adhesion molecule
FFPE: Formalin-fixed paraffin-embedded
GEP: Gene expression profiling
GSEA: Gene set enrichment analysis
iNOS1: Nitric oxide synthase, inducible 1
MpBC: Metaplastic breast carcinoma
MS4A4A: Membrane Spanning 4-Domains A4A
MSigDB: Molecular signatures database
NST: No special type
Olfr78: Omnibuslfactory receptor-78
PMA: Phorbol-12-myristate-13-acetate
qRT-PCR: quantitative reverse transcription polymerase chain reaction
ROI: Region of interest
RPMI: Roswell Park Memorial Institute
shRNA: Short hairpin RNA
SPS: Spindle carcinomatous
TAM: Tumor-associated macrophage
TGF-β1: Transforming growth factor-β1
TME: Tumor microenvironment
TNBC: Triple-negative breast cancer
TWIST1: Twist family bHLH transcription factor 1
VIM: Vimentin
ZEB1: Zinc finger E-box binding homeobox 1
ZEB2: Zinc finger E-box binding homeobox 2
