# Supplementary material for: Carcinomas exhibiting epithelial–mesenchymal transition manifest an M2 macrophage-enriched tumor immune microenvironment
Source: Breast Cancer Res. 2025 Oct 14;27:177. doi: 10.1186/s13058-025-02119-1 (PMC12522275; doi:10.1186/s13058-025-02119-1)
Supplement: Supplementary file 1 — Supplementary Material 1 [file 13058_2025_2119_MOESM1_ESM.docx]

**Supplementary Table S1** Primer sequences used in the quantitative real-time polymerase chain reaction

| Gene | Forward sequence | Reverse sequence |
| --- | --- | --- |
| *CD163* | GGCTCAATGAAGTGAAGTGCAAAG | CCAAGGATCCCGACTGCAA |
| *iNOS* | TCTCCGACCACCACTACAGCAA | GGGGAACTGGGCAGACTCAA |
| *CSF1* | GATGGAGACCTCGTGCCAAATTA | TGTTATCTCTGAAGCGCATGGTG |
| *GAPDH* | TCGGAGTCAACGGATTTGGT | TTCCCGTTCTCAGCCTTGAC |
