# Supplementary material for: Carcinomas exhibiting epithelial–mesenchymal transition manifest an M2 macrophage-enriched tumor immune microenvironment
Source: Breast Cancer Res. 2025 Oct 14;27:177. doi: 10.1186/s13058-025-02119-1 (PMC12522275; doi:10.1186/s13058-025-02119-1)
Supplement: Supplementary file 2 — Supplementary Material 2 [file 13058_2025_2119_MOESM2_ESM.docx]

**Supplementary Table S2.** Differentially expressed immune-oncological protein markers of paired spindle carcinomatous (SPS) versus no special type (NST) components in three cases of metaplastic breast carcinoma (MpBC)

| MBC6 (SPS versus NST) | |  |  |
| --- | --- | --- | --- |
|  | log2FC(SPS/NST) | P.Value | Annotation |
| CD20 | 0.7514 | 0.0016984 | B cell |
| PD-1 | 0.5407 | 0.0188774 | Checkpoint |
| CTLA4 | 0.894 | 0.0210718 | Checkpoint |
| CD66b | 2.5717 | 0.0003139 | Granulocyte |
| CD34 | 1.7418 | 0.0006532 | Hematopoietic |
| CD68 | 1.1774 | 0.0085634 | Myeloid/monocyte/macrophage |
| CD14 | 1.3739 | 0.0004914 | Myeloid/monocyte/macrophage |
| CD163 | 1.8584 | 0.0084282 | Myeloid/monocyte/macrophage |
| CD56 | 0.6627 | 0.0134138 | NK cell |
| CD3 | -0.5673 | 0.0158434 | T cell |
| CD127 | 0.7097 | 0.0004418 | T cell |
| CD25 | 0.9218 | 4.15E-05 | T cell |
| FOXP3 | 1.0916 | 0.0160798 | Treg |
| MBC24 (SPS versus NST) | |  |  |
|  | log2FC(SPS/NST) | P.Value | Annotation |
| CD14 | 2.0064 | 0.00018982 | Myeloid/monocyte/macrophage |
| PD-L2 | 1.7353 | 0.00294735 | Checkpoint |
| PD-L1 | 1.9016 | 0.00302954 | Checkpoint |
| GZMB | -1.7485 | 0.00306936 | T cell |
| CD8 | 0.9648 | 0.01232402 | T cell |
| CD27 | -0.9641 | 0.01417746 | T cell |
| CD4 | -0.6056 | 0.03593031 | T cell |
| MBC27 (SPS versus NST) | |  |  |
|  | log2FC(SPS/NST) | P.Value | Annotation |
| CD14 | 1.4810 | 0.00999913 | Myeloid/monocyte/macrophage |
| CD34 | 1.3887 | 0.00153244 | Hematopoietic |
| Ki-67 | 1.3585 | 0.00443989 | Proliferation |
| PD-L2 | 0.9854 | 0.0256518 | Checkpoint |
| HLA-DR | 0.9336 | 0.0031618 | Antigen presenting cell |
| CD163 | 0.8846 | 0.03334895 | Myeloid/monocyte/macrophage |
| CD11c | 0.8437 | 0.02142971 | Dendritic cell |
| CTLA4 | 0.7491 | 0.0296604 | Checkpoint |
| CD27 | -0.5555 | 0.03412088 | T cell |
