# Supplementary material for: Carcinomas exhibiting epithelial–mesenchymal transition manifest an M2 macrophage-enriched tumor immune microenvironment
Source: Breast Cancer Res. 2025 Oct 14;27:177. doi: 10.1186/s13058-025-02119-1 (PMC12522275; doi:10.1186/s13058-025-02119-1)
Supplement: Supplementary file 3 — Supplementary Material 3 [file 13058_2025_2119_MOESM3_ESM.docx]

**Supplementary Table S3.** IO360-defined immune cell type scores for the no special type (NST) and paired spindle carcinomatous (SPS) components in six metaplastic breast carcinoma (MpBC) cases.

|  | MBC5-NST | MBC5-SPS | MBC15-NST | MBC15-SPS | MBC42-NST | MBC42-SPS | MBC91-NST | MBC91-SPS | MBC95-NST | MBC95-SPS | MBC122-NST | MBC122-SPS |
| --- | --- | --- | --- | --- | --- | --- | --- | --- | --- | --- | --- | --- |
| Cytotoxic cells | 4.6432 | 4.5879 | 5.8316 | 4.6816 | 3.9145 | 6.1574 | 5.2917 | 6.5767 | 6.1227 | 5.6074 | 5.5288 | 5.5961 |
| Macrophages | 7.7471 | 9.0878 | 7.9049 | 10.4594 | 6.8714 | 10.5095 | 7.5838 | 9.3163 | 7.8680 | 9.4313 | 9.4084 | 10.3311 |
| Dendritic cells | 4.1436 | 4.5543 | 4.7429 | 6.0111 | 3.4951 | 7.2397 | 3.5722 | 5.4573 | 5.0089 | 5.5656 | 6.1398 | 5.8643 |
| Exhausted CD8 | 5.8689 | 5.9405 | 5.7031 | 4.9302 | 5.4972 | 6.8817 | 6.0452 | 6.8640 | 6.5607 | 6.6913 | 5.4056 | 5.8533 |
| B-cells | 4.2725 | 4.2268 | 4.4682 | 4.2391 | 3.9705 | 5.5904 | 4.3891 | 5.6861 | 5.4613 | 4.3895 | 4.7556 | 4.1097 |
| CD45 | 5.7232 | 6.0414 | 6.4271 | 6.6968 | 5.0019 | 7.3879 | 5.7951 | 6.9183 | 6.9024 | 6.7644 | 6.6033 | 6.8640 |
| Treg | 5.6130 | 5.6678 | 6.1395 | 4.5934 | 5.6357 | 6.3726 | 4.9418 | 6.0485 | 6.3511 | 6.0096 | 6.3308 | 6.1426 |
| Neutrophils | 5.8009 | 6.0055 | 5.6168 | 6.5031 | 4.9180 | 7.4412 | 5.1486 | 6.0772 | 5.3845 | 6.3443 | 6.4473 | 6.0580 |
| T-cells | 4.8278 | 4.3830 | 5.8313 | 4.2263 | 4.6920 | 6.5411 | 5.6575 | 6.3560 | 6.1956 | 5.5604 | 5.6022 | 5.1506 |
| NK CD56dim cells | 4.2316 | 4.2294 | 4.6922 | 3.3146 | 3.7778 | 4.4444 | 3.9696 | 4.3768 | 4.3475 | 2.4105 | 4.2409 | 3.1241 |
| Mast cells | 4.9907 | 5.5560 | 6.1148 | 6.1567 | 4.8846 | 8.2495 | 3.8765 | 4.4087 | 5.6646 | 5.7293 | 6.9536 | 6.7766 |
| CD8 T cells | 5.2666 | 4.7295 | 7.1924 | 5.1974 | 5.2801 | 7.3653 | 6.7238 | 7.4217 | 7.3836 | 6.1312 | 6.0674 | 6.0010 |
| NK cells | 3.9135 | 3.2970 | 4.3881 | 4.0174 | 4.0305 | 5.4195 | 3.7614 | 4.4400 | 5.6615 | 5.5272 | 5.1360 | 4.0030 |
| Th1 cells | 4.2396 | 4.6947 | 5.0310 | 3.8565 | 4.1951 | 5.6356 | 4.6448 | 4.6741 | 4.4404 | 4.5874 | 3.9574 | 3.8888 |
