# Supplementary material for: Carcinomas exhibiting epithelial–mesenchymal transition manifest an M2 macrophage-enriched tumor immune microenvironment
Source: Breast Cancer Res. 2025 Oct 14;27:177. doi: 10.1186/s13058-025-02119-1 (PMC12522275; doi:10.1186/s13058-025-02119-1)
Supplement: Supplementary file 4 — Supplementary Material 4 [file 13058_2025_2119_MOESM4_ESM.docx]

**Supplementary Table S4** Gene expression levels of inducing cytokines and cell markers for M1 and M2 macrophages in paired spindle carcinomatous (SPS) and no special type (NST) components across six metaplastic breast carcinoma (MpBC) cases

|  |  |  | NST components | |  |  |  |  |  | SPS components | |  |  |
| --- | --- | --- | --- | --- | --- | --- | --- | --- | --- | --- | --- | --- | --- |
|  | MBC122 | MBC15 | MBC42 | MBC5 | MBC91 | MBC95 |  | MBC122 | MBC15 | MBC42 | MBC5 | MBC91 | MBC95 |
| M1 macrophage | |  |  |  |  |  |  |  |  |  |  |  |  |
| CD86 | 111 | 58 | 39 | 70 | 84 | 77 |  | 163 | 160 | 126 | 103 | 54 | 389 |
| CSF2 | 28 | 22 | 26 | 21 | 24 | 25 |  | 18 | 8 | 49 | 17 | 25 | 16 |
| IFNG | 9 | 16 | 20 | 32 | 43 | 43 |  | 16 | 14 | 25 | 21 | 46 | 25 |
| TNF | 91 | 171 | 68 | 941 | 164 | 66 |  | 25 | 31 | 75 | 154 | 52 | 38 |
| NOS2 | 22.17 | 25.58 | 8.91 | 48.28 | 10.43 | 18.05 |  | 34.62 | 22.2 | 19.84 | 42.46 | 12.51 | 24.11 |
|  |  |  |  |  |  |  |  |  |  |  |  |  |  |
| M2 macrophage | |  |  |  |  |  |  |  |  |  |  |  |  |
| CD163 | 584 | 454 | 240 | 267 | 307 | 277 |  | 2053 | 1434 | 1537 | 804 | 890 | 1691 |
| CD84 | 262 | 124 | 88 | 165 | 96 | 129 |  | 466 | 513 | 298 | 395 | 339 | 162 |
| IL10 | 14 | 14 | 18 | 17 | 25 | 54 |  | 33 | 69 | 39 | 38 | 29 | 80 |
| CD206 | 263 | 44 | 45 | 56 | 39 | 91 |  | 323 | 297 | 294 | 59 | 138 | 128 |
| TGFB1 | 1121 | 1441 | 1151 | 2411 | 1269 | 1254 |  | 3475 | 3599 | 2679 | 7703 | 2748 | 3965 |
| TGFB2 | 532 | 304 | 937 | 125 | 41 | 306 |  | 528 | 265 | 231 | 109 | 113 | 1128 |
| TGFB3 | 313 | 1020 | 1108 | 1068 | 1507 | 371 |  | 4733 | 8006 | 1640 | 8467 | 3869 | 384 |
