# Supplementary material for: Carcinomas exhibiting epithelial–mesenchymal transition manifest an M2 macrophage-enriched tumor immune microenvironment
Source: Breast Cancer Res. 2025 Oct 14;27:177. doi: 10.1186/s13058-025-02119-1 (PMC12522275; doi:10.1186/s13058-025-02119-1)
Supplement: Supplementary file 7 — Supplementary Material 7 [file 13058_2025_2119_MOESM7_ESM.docx]

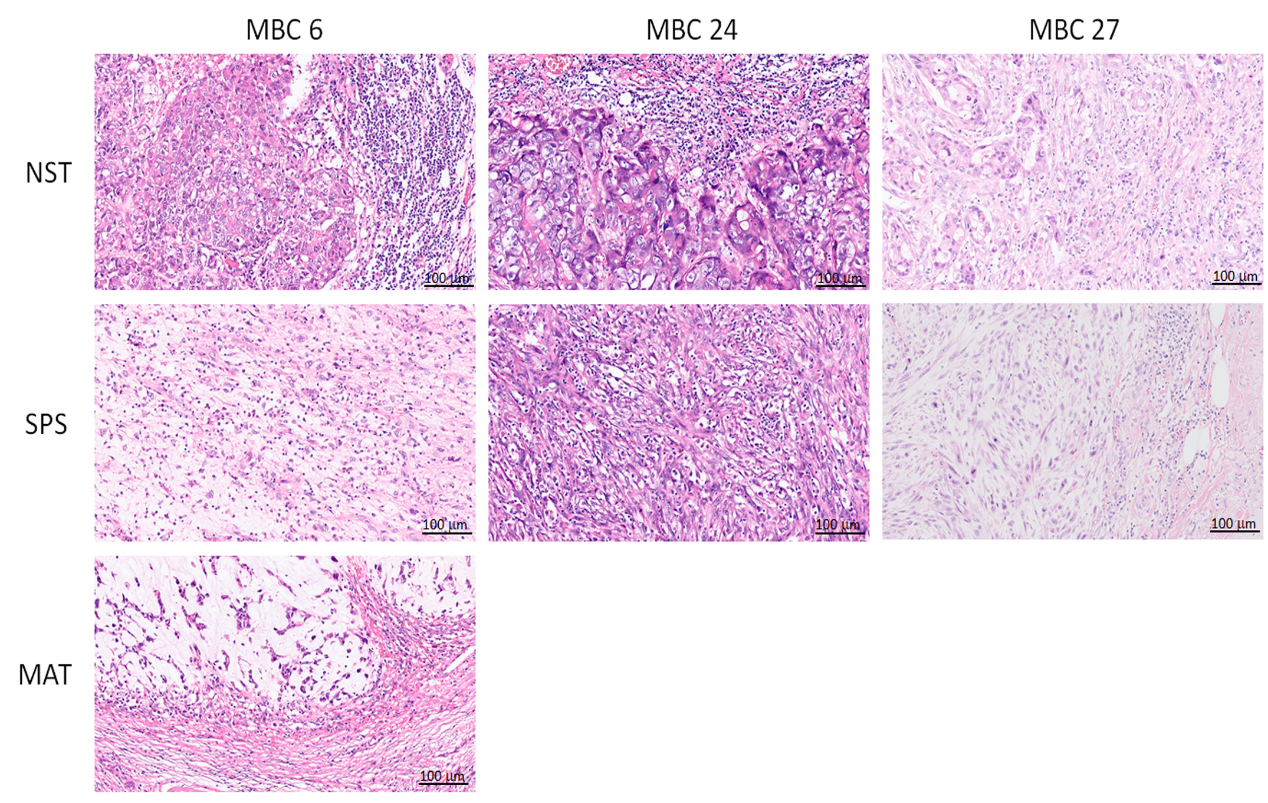


**Supplementary Figure S1** Histomorphology of the three metaplastic breast carcinoma (MpBC) cases (MBC6, MBC24, and MBC27) as indicated by GeoMx Digital Spatial Profiler protein assay. Carcinoma with no special type (NST) components and spindle carcinomatous (SPS) components are present in all three MpBC cases. MBC6 exhibits an additional matrix-producing (MAT) component.
