# Supplementary material for: Carcinomas exhibiting epithelial–mesenchymal transition manifest an M2 macrophage-enriched tumor immune microenvironment
Source: Breast Cancer Res. 2025 Oct 14;27:177. doi: 10.1186/s13058-025-02119-1 (PMC12522275; doi:10.1186/s13058-025-02119-1)
Supplement: Supplementary file 8 — Supplementary Material 8 [file 13058_2025_2119_MOESM8_ESM.docx]

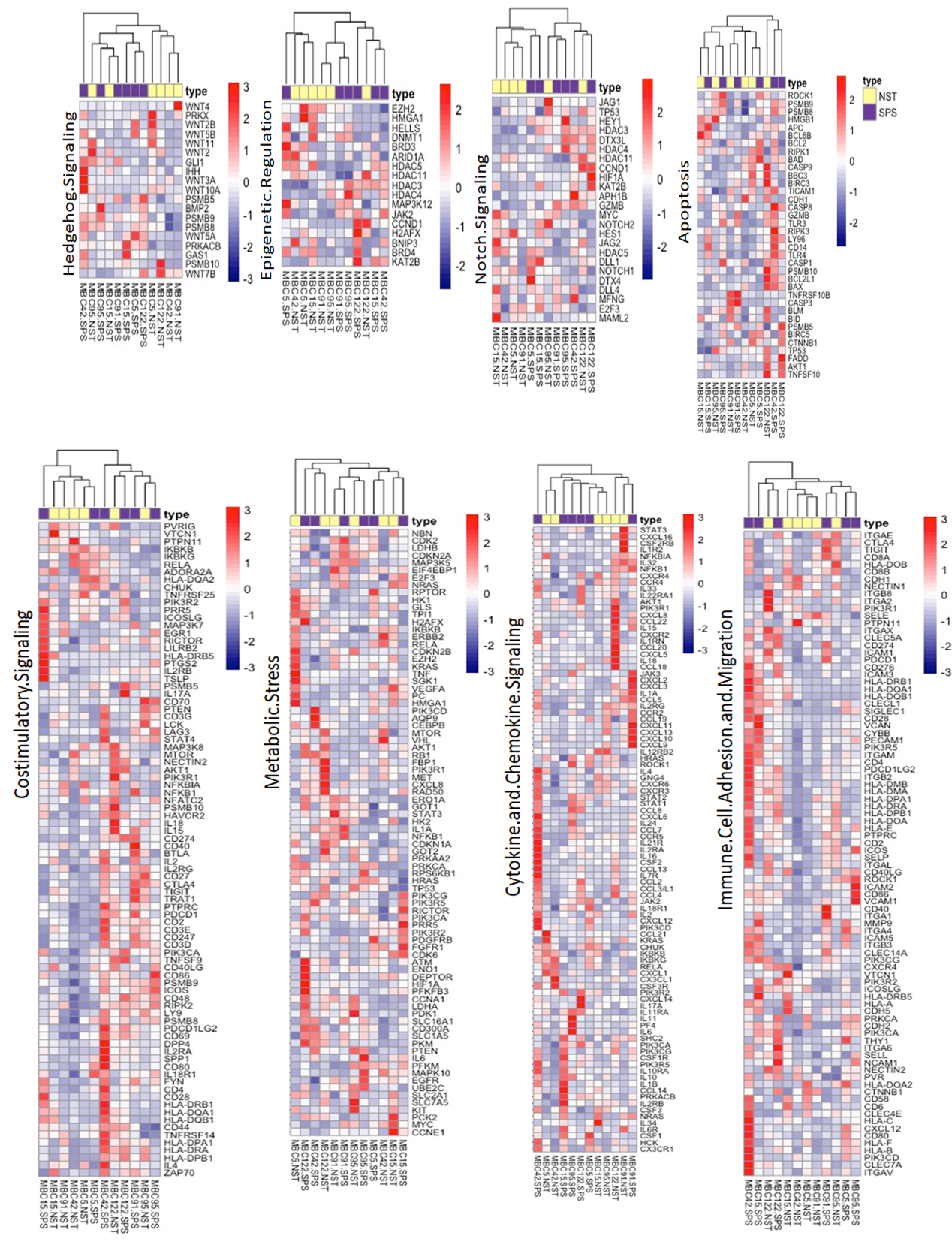


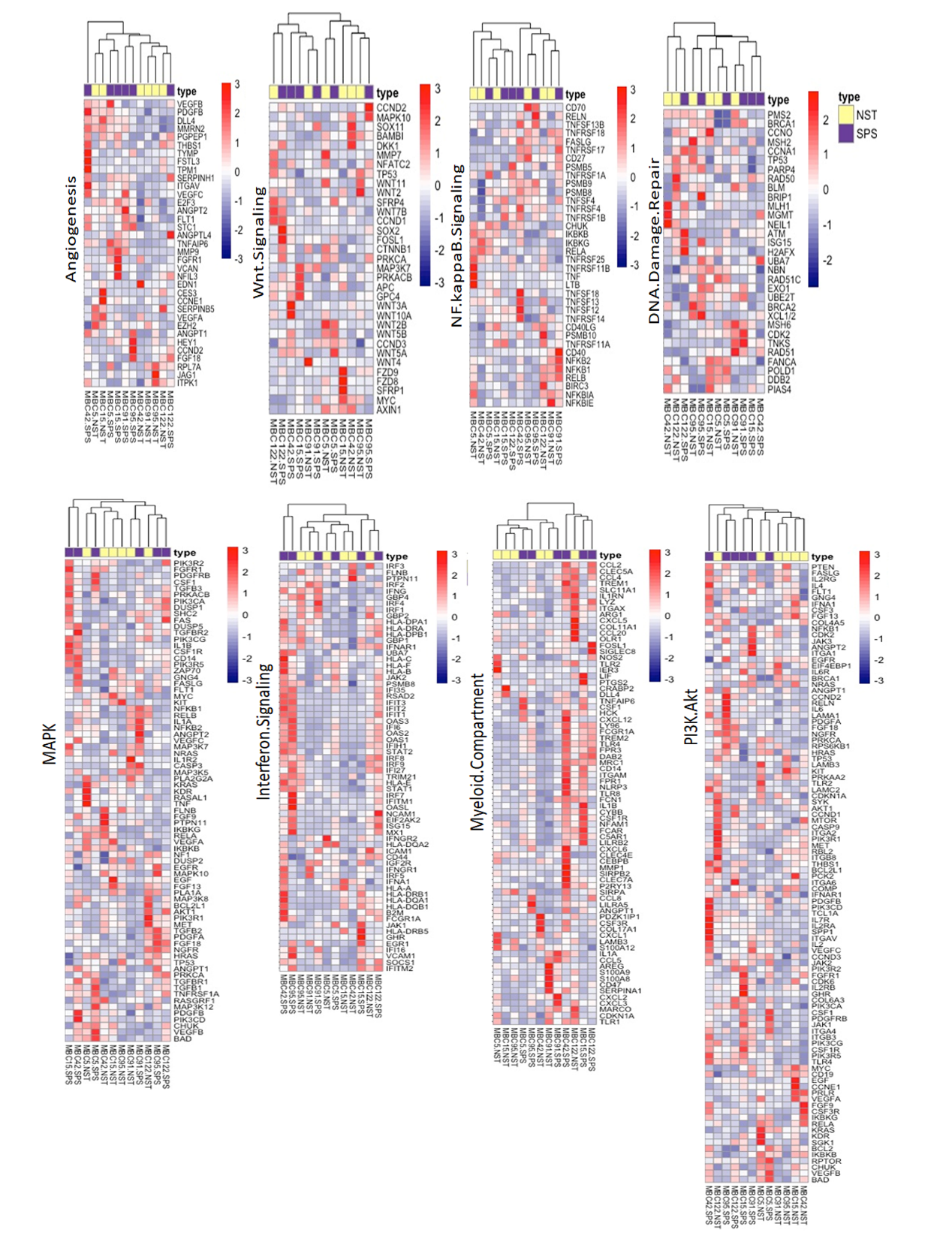


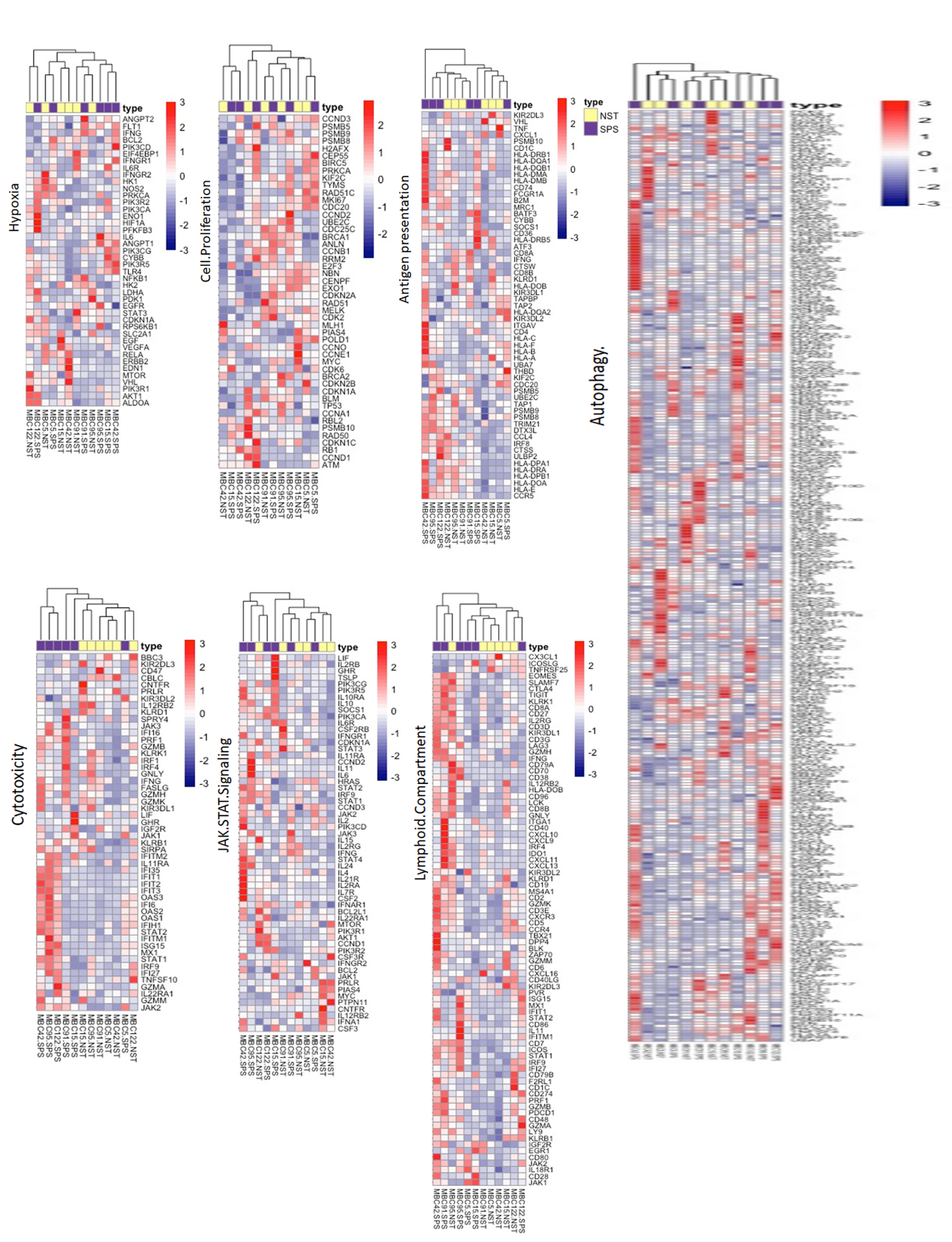


**Supplementary Figure S2** Heatmaps illustrating NanoString-defined biological pathways observed in the paired spindle carcinomatous (SPS) and paired no special type (NST) components of the six metaplastic breast carcinoma (MpBC) cases
