# Supplementary figures and images for: Carcinomas exhibiting epithelial–mesenchymal transition manifest an M2 macrophage-enriched tumor immune microenvironment
Source: Breast Cancer Res. 2025 Oct 14;27:177. doi: 10.1186/s13058-025-02119-1 (PMC12522275; doi:10.1186/s13058-025-02119-1)

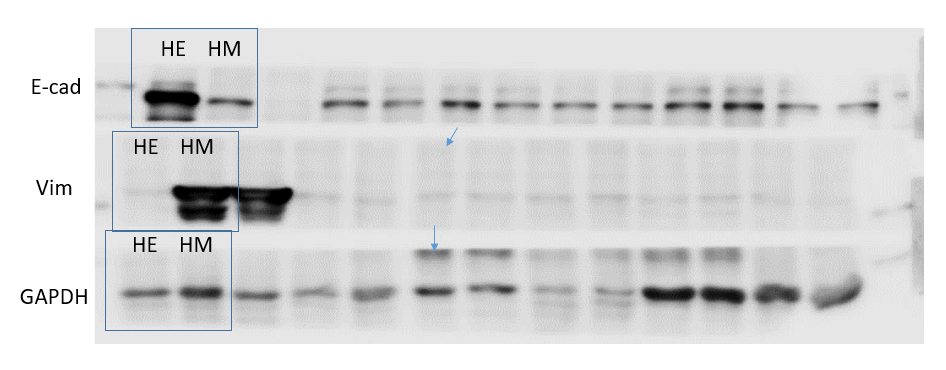


**Supplementary Fig. S3** Full uncropped Gels and Blots image of Fig. 5A

Supplement: Supplementary file 9 — Supplementary Material 9 [file 13058_2025_2119_MOESM9_ESM.docx]

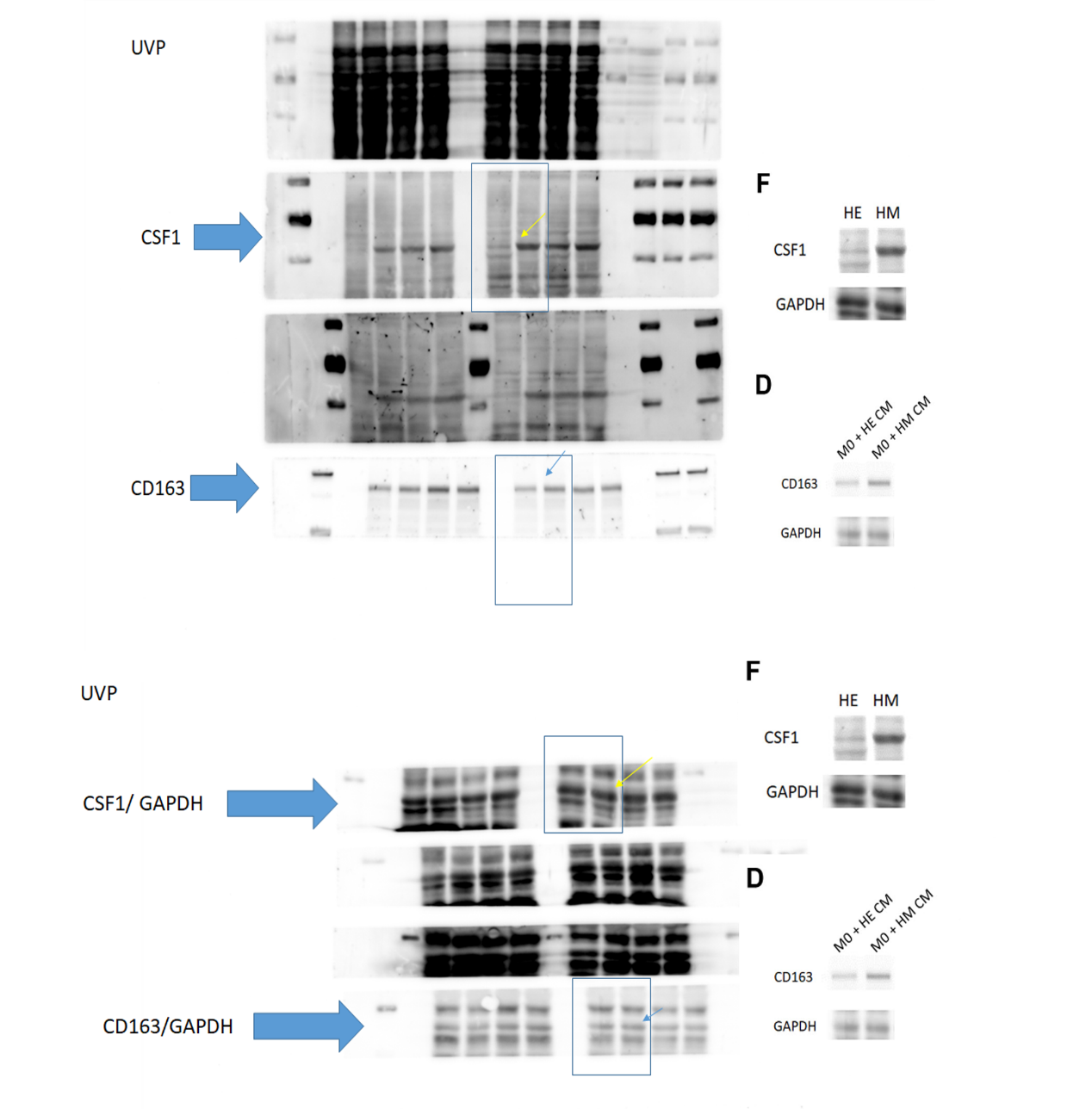


**Supplementary Fig. S4** Full uncropped Gels and Blots image of Fig. 5D and 5F.

Supplement: Supplementary file 10 — Supplementary Material 10 [file 13058_2025_2119_MOESM10_ESM.docx]
