# Supplementary material for: Carcinomas exhibiting epithelial–mesenchymal transition manifest an M2 macrophage-enriched tumor immune microenvironment
Source: Breast Cancer Res. 2025 Oct 14;27:177. doi: 10.1186/s13058-025-02119-1 (PMC12522275; doi:10.1186/s13058-025-02119-1)
Supplement: Supplementary file 11 — Supplementary Material 11 [file 13058_2025_2119_MOESM11_ESM.docx]

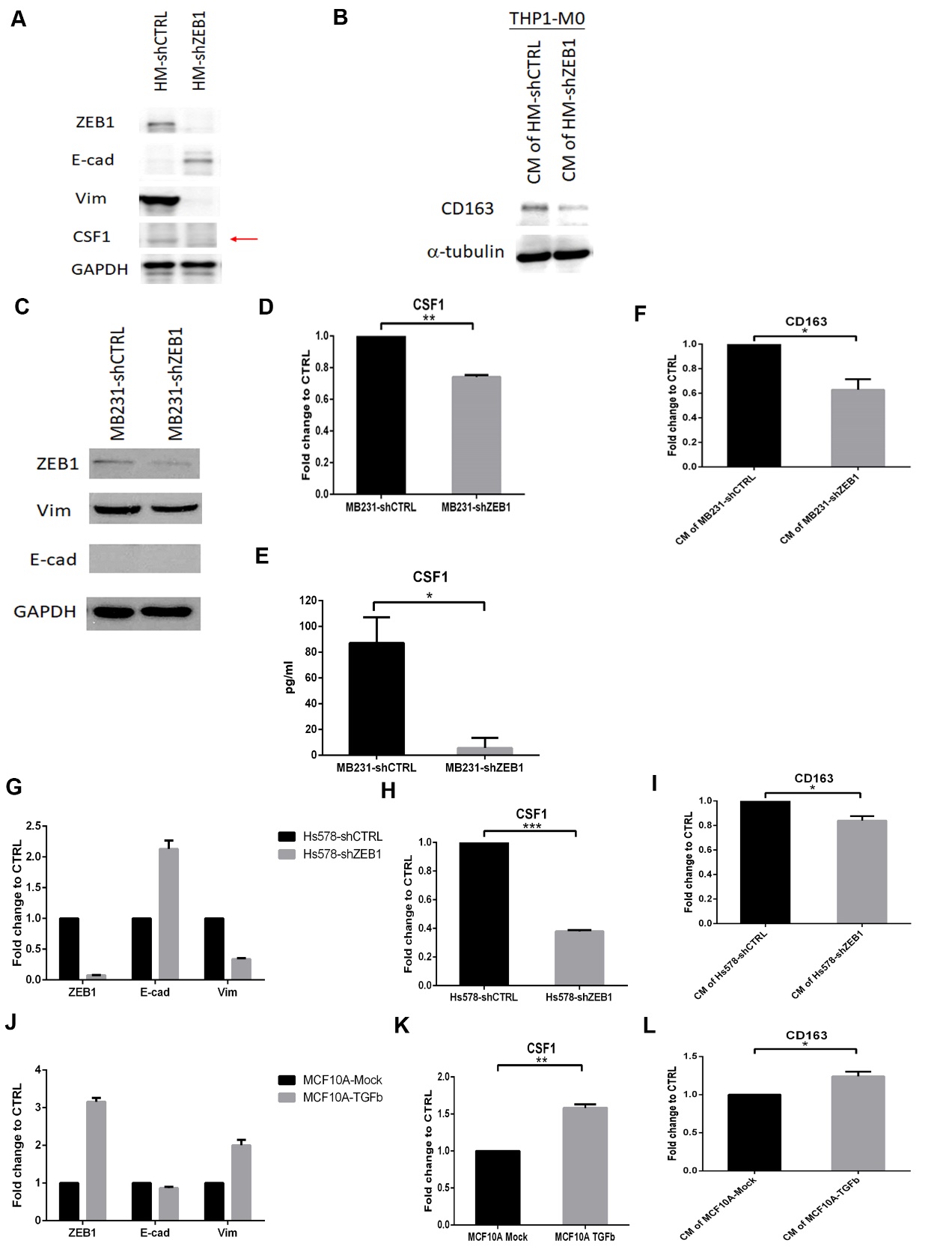


**Supplementary Figure 5** Epithelial–mesenchymal transition (EMT) enhances the ability of breast cancer cells to promote M2 macrophage polarization. **A.** Western blot analysis of ZEB1, E-cadherin (E-cad), vimentin (Vim), and CSF1 in HM cells with ZEB1 knockdown (HM-shZEB1) versus control (HM-shCTRL). **B.** Western blot showing CD163 expression, an M2 macrophage marker, in THP1-derived M0 macrophages treated with conditioned media (CM) from HM-shZEB1 or HM-shCTRL cells. **C.** Western blot analysis of ZEB1, E-cad, and Vim in MDA-MB-231 cells with ZEB1 knockdown (MB231-shZEB1) compared to control (MB231-shCTRL). **D.** Bar graph showing fold change in CSF1 mRNA expression (qRT-PCR**)** in MB231-shZEB1 versus MB231-shCTRL cells. **E.** Bar graph showing fold change in **CSF1** protein expression (ELISA**)** in CM from MB231-shZEB1 versus MB231-shCTRL cells. **F.** Bar graph showing fold change in **CD163** mRNA expression (qRT-PCR**)** in THP1-derived M0 macrophages treated with CM from MB231-shZEB1 versus MB231-shCTRL cells. **G, H.** Bar graphs showing fold changes in **ZEB1**, **E-cad**, and **Vim** (**G**) and **CSF1** (**H**) expression in Hs 578T cells with ZEB1 knockdown (Hs 578T-shZEB1) versus control (Hs 578T-shCTRL). **I.** Bar graph showing fold change in **CD163** expression in THP1-derived M0 macrophages treated with CM from Hs 578T-shZEB1 versus Hs 578T-shCTRL cells. **J, K.** Bar graphs showing fold changes in **ZEB1**, **E-cad**, **Vim** (**J**) and **CSF1** (**K**) expression in MCF10A cells treated with TGF-β1 (5 ng/mL for 120 hours) compared to vehicle control. **L.** Bar graph showing fold change in **CD163** expression in THP1-derived M0 macrophages treated with CM from TGF-β1–treated MCF10A cells versus control. *p < 0.05; **p < 0.01; unpaired t-test.
